# Boerhaave’s syndrome after pentazocine-induced vomiting in a 21-year-old male with asthma: a case report

**DOI:** 10.11604/pamj.2021.38.74.27031

**Published:** 2021-01-21

**Authors:** Oyindamola Ibukun Awofisoye, Olaleye Emmanuel Olalekan, Ndubuisi Anumenechi, Frankilin Onwukpa

**Affiliations:** 1 Cardiocare Specialty Hospital, Limi Hospital, Abuja, Nigeria

**Keywords:** Boerhaave’s, pneumomediastinum, oesophageus, case report

## Abstract

Boerhaave's syndrome is an uncommon syndrome characterized by spontaneous rupture of the oesophagus with a high mortality rate. While excessive alcohol intake and binge-eating are the classic precipitants of this syndrome, medication-induced vomiting causing Booerhave's is quite uncommon. Traditionally managed operatively, conservative management is being increasingly reported in selected cases. We report the case of 21-year-old male with who developed sudden onset chest pain and dyspnoea after pentazocine induced vomiting. He was referred after lack of response to initial treatment for acute severe asthma. A chest CT scan showed pneumomediastinum, subcutaneous emphysema and oesophageal tear. He was managed conservatively with oxygen therapy, nil per mouth and antibiotics with improvement of symptoms and discharge after 8 days.

## Introduction

Spontaneous rupture of the oesophagus associated with forceful vomiting (Boerhaave's syndrome) is an uncommon disease of the gastrointestinal tract, but with a very high mortality rate. The rupture is longitudinal and transmural. The syndrome was first described in 1724 by the German doctor, Hermann Boerhaave. It classically followed excessive alcohol intake and vomiting [1]. Other triggers are less common and include binge-eating and caustic ingestion. Boerhaave´s syndrome complicating medication-induced vomiting is rare with only a handful of cases in the literature. The classic symptoms constitutes the Mackler´s triad: vomiting, lower thoracic pain and subcutaneous emphysema [2]. Because of its rarity, the diagnosis can easily be missed or delayed, leading to complications like mediastinitis, sepsis and shock. In such situations, the mortality can be very high [3]. We report the occurrence of Boerhaave´s syndrome in a 21-year-old Nigerian male which developed after pentazocine-induced vomiting.

## Patient and observation

The patient was a 21-year-old male, known to have mild intermittent asthma. He was being treated for malaria at another centre after he presented with headaches and myalgia. He initially had parenteral antimalarials, but after pentazocine was administered, he vomited forcefully three times with retching. Shortly after vomiting, he developed a worsening retrosternal pain, with cough, dyspnoea and dysphagia. He was thought to have acute severe asthma and he was treated by nebulization with salbutamol, steroids and antibiotics but he continued to deteriorate with respiratory distress and oxygen saturation falling to about 80-85%, requiring supplemental oxygen therapy. This necessitated referral to our facility. On evaluation here, further history revealed that he has not had any asthma symptoms in 3 years nor required inhaler use. Additionally, the chest pain was retrosternal and markedly pleuritic.

Examination revealed a young man in respiratory distress on oxygen therapy. He was neither pale nor cyanosed. His respiratory rate was 28 breaths per minute. He was without wheeze or stridor. The percussion notes were hyper-resonant in the in the left hemithorax medially. The breath sounds were normal without rhonchi. He had mild chest wall tenderness. Abdominal examination was unremarkable. The pulse rate on admission was 106/min, regular with normal volume. The blood pressure was 130/88mmHg, while he required oxygen at 4 litres per minute to keep the SpO2 above 94%. His temperature was 37.6 degrees celsius, though was normal after the first day of admission.

**Figure 1:**
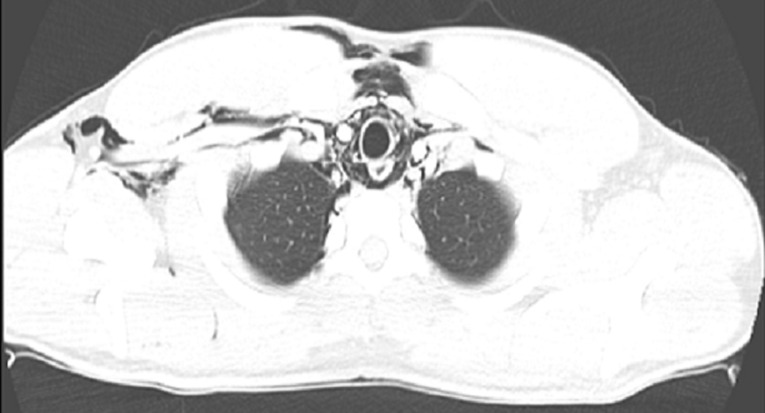
chest CT scan: axial view showing pneumomediastinum and subcutaneous emphysema

**Figure 2:**
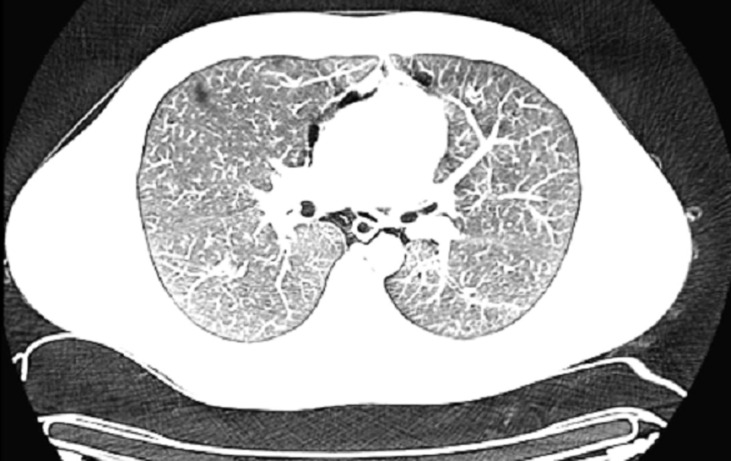
chest CT scan: axial view showing pneumomediastinum and a thin rim of pneumothorax

**Figure 3:**
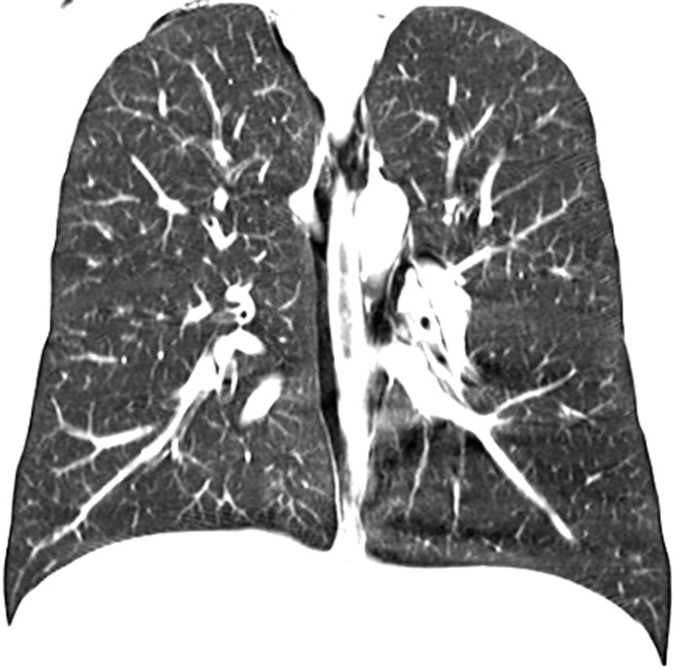
chest CT scan: coronal view showing air pneumomediastinum and discontinuity in the oesophageal wall about 2cm from the gastroesophageal junction

A possible spontaneous pneumothorax complicating resolved acute severe asthma was considered. An urgent chest CT scan was requested (Figure 1, Figure 2, Figure 3) which revealed a pneumomediastinum, a patulous oedematous oesophagus with an area of discontinuity about 2cm to the gastroesophageal junction (Figure 3). There was also right cervical subcutaneous emphysema (Figure 1). A diagnosis of Boerhaave´s syndrome was made about 24 hours after presentation. The blood count showed relative neutrophilia and the d-dimer was elevated. Other investigations done including, erythrocyte sedimentation rate, troponin T, SARS-CoV-2 test and electrocardiography were normal (Table 1).

**Table 1:** laboratory results

Parameter	Value	Reference range
**Complete blood count**		
Hemoglobin (g/dl)	15.8	13.4 - 17.5
Total white cell count (x10^6^/L)	9,300	3,900 - 10,400
Neutrophil %	90.1%	32 - 76%
Lymphocytes %	7.2%	18 - 56%
Platelet count (x10^9^/L)	120	150 - 400
**E/U/Cr**		
Sodium (mmol/L)	140	135 - 145
Potassium (mmol/L)	4.6	3.0 - 5.2
Chloride (mmol/L)	94	95 - 105
Bicarbonate (mmol/L)	25	20 - 30
Urea (mg/dl)	17	15 - 50
Creatinine (mg/dl)	1.0	0.5 - 1.2
Erythrocyte sedimentation rate (mm/hr)	23	<30
D-dimer (ng/ml)	1760	0 - 500
Troponin-T (ng/l)	<50	<50
SARS-CoV-2 PCR	Negative	

**Figure 4:**
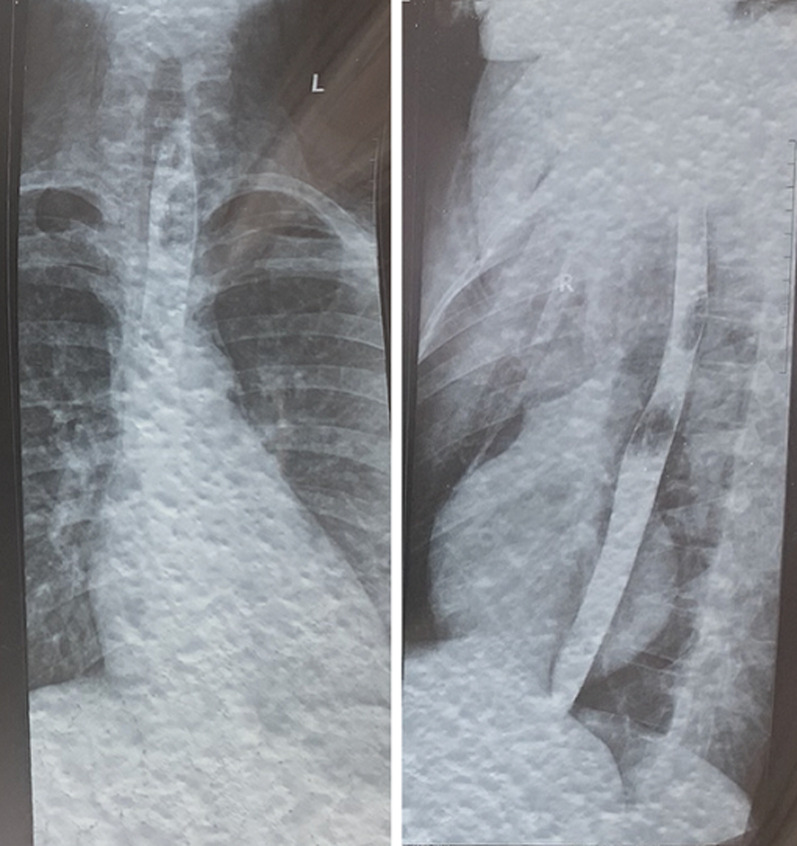
barium swallow (eighth day) anteroposterior and lateral views showing no barium spillage from the oesophagus

By the second day of admission the subcutaneous emphysema was clinically apparent on the anterior chest wall, extending to the supraclavicular fossa and lower neck. However, his respiratory rate and chest pain score were improving. He was managed conservatively with intravenous antibiotics, nil per oral, intravenous fluids, total parenteral nutrition and supplemental oxygen. By day 4, his symptoms were mostly resolved with mild residual retrosternal pain. He had a barium swallow on day 7 of admission which did not show any leakage from the oesophagus (Figure 4). Graded oral feeding was started on day 8 and he was discharged to ambulatory care. The subcutaneous emphysema resolved after two weeks.

## Discussion

Majority of oesophageal perforations are iatrogenic, with only about 15% being spontaneous [4]. While Booerhave's syndrome is often described as a “spontaneous” rupture, it is due to a rise in the intraluminal pressure of the oesophagus. The classic triad includes forceful vomiting, chest pain and subcutaneous emphysema. Only about three cases are reported per million persons per year with most being middle-aged persons and above. Our case is unusual as it is uncommon in children and adolescents. The typical triggers are excessive alcohol consumption and large intake of meals. Medication-induced vomiting causing Boerhaave´s syndrome is quite uncommon and no previous report for pentazocine was found in the literature. The commonest location of the tear is at the left posterolateral wall of the lower third of the esophagus 2-3cm proximal to the GEJ along the longitudinal wall. The intrathoracic perforation can lead to chemical mediastinitis, necrosis and bacterial infection. It is unclear why our patient did not develop significant mediastinitis and could be managed conservatively. Possible reasons include: minimal length of the tear; the stomach was relatively empty due to poor feeding preceding the vomiting; it was not preceded by alcohol intake or binge eating; steroids were used to treat the supposed “asthma exacerbation” which could have inadvertently limited any inflammation.

The clinical manifestations of Boerhaave syndrome depends on the location of the rupture, the extent and the time from onset of rupture. The symptoms are often non-specific and diagnosis is often missed or delayed like in our patient. The history of vomiting preceding chest symptoms can be suggestive, although hematemesis is only occasionally present. Subcutaneous emphysema is observed within the first 24 hours in 28-60% of cases [5], though in our patient it was only noticed after 48 hours. Auscultation may also reveal a crackling sound heard with the heart beats (Hamman crunch) that signifies pneumomediastinum which is reported in about 20% of cases [5]. This was absent in our patient, despite the large amount of pneumomediastinum on CT scan. Chest finding are more often on the left because 90% of the tears are in the left posterolateral wall of the oesophagus, which communicates with the left pleural cavity in most cases [3]. The suspicion of pneumothorax in our patient was due to the sudden clinical presentation and finding of hyper-resonant percussion notes.

Sudden onset shortness of breath and chest pain in someone with asthma should make one think of a spontaneous pneumothorax; which was the main reason for urgent imaging in this patient. However, other differential diagnosis should be considered, especially when the usual clinical signs of acute severe asthma were not present like in our patient. Booerhave´s syndrome has also been misdiagnosed as acute aortic syndrome, pericarditis, pulmonary embolism or spontaneous pneumothorax [6] in other reports. Chest X-ray is usually the initial imaging modality which is abnormal in majority of patients. The usual abnormalities reported include left-sided pleural effusion, pneumothorax, subcutaneous emphysema and pneumomediastinum which manifests as V-sign described by Naclerio [5]. Chest CT scan was the initial modality used in this case in view of the higher diagnostic yield and the severity of symptoms.

Unenhanced chest CT scan findings may include the aforementioned findings, additionally peri-oesophageal air collections, intramural hematoma and oesophageal wall thickening amongst others [7]. The most consistent findings are peri-oesophageal air collection, pneumomediastinum and subcutaneous emphysema which were present in our patient. CT scan is however limited in precisely defining the tear compared to oesophagography. Esophagogram is performed using barium or gastrograffin and it typically shows contrast extravasation at a supradiaphragmatic level. However, this was delayed in this patient as the diagnosis was already made from the CT scan and we believed the risk associated with extravasation of contrast medium was avoidable. It was normal on the eight day, indicating that healing has occurred. The negative test was reassuring in recommencement of oral feeding. It is noteworthy that false negative tests has been reported in contrast studies for oesophageal perforation [8].

The traditional management of Booerhaves syndrome is surgical. But there are several reports of successful conservative (non-operative) management [9-11]. The factors which support conservative management as suggested by Cameron *et al*. includes: oesophageal disruption well contained in the mediastinum; any cavity should be well drained back into the oesophagus; minimal symptoms, and minimum evidence of sepsis [12]. These were met in this patient. Conservative management in this patient included nil by mouth, intravenous fluids and antibiotics. Nasogastric tube drainage and thoracostomy are also components of conservative care, but was not necessary in this patient as mediastinal leakage was mostly air and inflammatory symptoms were minimal.

## Conclusion

Medication induced vomiting may be complicated by oesophageal tear and a high index of suspicion is required in patients that develop sudden onset chest pain and dyspnoea after such vomiting. The differential diagnosis of chest pain and dyspnoea is vast and imaging is critical for establishing a diagnosis in ambiguous cases. Conservative management of mild cases of Booerhave´s syndrome is possible with early diagnosis and prompt treatment.
